# Prevention of Air Embolism in Extracorporeal Membrane Oxygenation Systems: An In Vitro Study on Protection of Central Venous Catheter Lumen

**DOI:** 10.3390/medicina60111883

**Published:** 2024-11-17

**Authors:** Danilo Franco, Nejc Krasna, Robert Novak, Giovanni Esposito, Raffaele Izzo, Jan Belohlavek, Marko Noc, Tomaz Goslar

**Affiliations:** 1 Center for Intensive Internal Medicine, University Medical Center, Zaloska 7, 1000 Ljubljana, Slovenia; marko.noc@mf.uni-lj.si (M.N.); tomaz.goslar@gmail.com (T.G.); 2 Department of Advance Biomedical Sciences, Federico II University, Via Pansini 5, 80131 Naples, Italy; giovanni.esposito2@unina.it (G.E.); rafizzo@unina.it (R.I.); 3 2nd Department of Medicine, Department of Cardiovascular Medicine, First Faculty of Medicine, Charles University in Prague and General University Hospital, 10000 Prague, Czech Republic; jan.belohlavek@vfn.cz; 4 Emergency Center, General Hospital Celje, Oblakova 5, 3000 Celje, Slovenia; krasna.nejc@gmail.com; 5 Department of Cardiovascular Surgery, University Medical Center, Zaloska 7, 1000 Ljubljana, Slovenia; novakrobert20@gmail.com; 6 Faculty of Medicine, University of Ljubljana, Vrazov trg 2, 1000 Ljubljana, Slovenia

**Keywords:** extracorporeal membrane oxygenation, air embolism, complications, simulation

## Abstract

*Background and Objectives:* This study aimed to investigate the risk and mechanisms of air entry into the extracorporeal membrane oxygenation (ECMO) circuit through the central venous catheter (CVC) in a veno-venous configuration. The primary goal was to assess the impact of different air volumes on ECMO circuit performance at varying pump speeds. *Material and Methods:* The study utilized a circuit model to simulate ECMO conditions and evaluate the potential entry points of air, specifically through the unprotected lumen of the CVC. Various interventions, such as the use of a closed three-way stopcock or clave, were implemented to assess their efficacy in preventing air entry. *Results:* The unprotected lumen of the central venous catheter posed a significant risk for air entry into the ECMO circuit. The introduction of a closed three-way stopcock or clave proved effective in preventing air ingress through the central venous catheter. Auditory cues, such as a distinct hissing sound, served as an early warning sign of air presence in the circuit. The study demonstrated that even small volumes of air, as minimal as 1 mL, could pass through the oxygenator at specific pump speeds, and larger volumes could lead to pump dysfunction. *Conclusions:* The study identified the unprotected lumen of the central venous catheter as a potential entry point for air into the ECMO circuit. The use of a closed three-way stopcock or one-way valve was found to be a reliable protective measure against air infiltration. Early detection through the observation of a hissing sound in the circuit provided a valuable warning sign. These findings contribute to enhancing the safety and performance of ECMO systems by minimizing the risk of air embolism.

## 1. Introduction

Over the past decade, extracorporeal membrane oxygenation (ECMO) has emerged as a critical life-saving intervention that provides cardiopulmonary support to patients. Despite significant advances in ECMO technology, such as heparin-coated circuits, flow probes, and downsized circuits, certain complications still occur with its use. Bleeding, emboli, infection, accidental decannulation, and air emboli (AE) are among the consequences that must be recognized and treated as soon as possible. The incidence of AE in patients treated with ECMO is up to 4.6% in adults [1] and up to 4.9% in neonates, indicating a small but significant risk [2]. Although AE is rare in patients treated with ECMO, it is very important that it be recognized quickly [2,3]. Animal studies suggest that the lethal dose of air in adults is between 200 and 300 mL or 3–5 mL/kg, causing circulatory collapse or tissue infarction, primarily obstructing venous blood flow in the lungs [4]. In ECMO-assisted patients, air can enter the circulation through damaged tubing, venous cannula disconnection or deconnections in venous cannulas, or through intravascular access point such as a central venous catheter (CVC). Additionally, broncho-venous fistulas resulting from high positive pressure during ventilation or percutaneous tracheostomy procedures have been identified as potential sources of AE [5]. The severe negative pressure in the venous system and the high blood flows mean that air can enter the systemic circulation even more rapidly during ECMO [6].

To mitigate this risk, various preventive strategies have been established to reduce the likelihood of air embolism during ECMO support. Routine circuit integrity checks are critical; all ECMO tubing, connections, and cannulas should be carefully inspected for damage or disconnection prior to ECMO initiation and monitored regularly throughout support to identify potential breaches early. Additionally, protective devices such as bubble detectors and alarms can rapidly identify the presence of air in the ECMO circuit, enabling prompt intervention to prevent air from reaching the patient.

Clamping protocols and closed infusion systems are also recommended to maintain a sealed environment, particularly during drug administration or circuit maintenance. This involves using clamping protocols to minimize accidental air entry, while closed infusion systems help further reduce the risk of introducing air into the circuit. For central venous catheters, which can be a potential entry point for air, the use of specialized connectors—such as claves and closed three-way stopcocks—can effectively prevent air from entering through catheter ports when not in use, maintaining a closed system that reduces the risk of air entry.

Additional preventive measures include careful procedural protocols for CVC insertion and manipulation. When feasible, CVC insertion at sites distant from ECMO cannula placement, such as the femoral site, is preferred to minimize the risk of air entrainment. During invasive procedures near the ECMO circuit, temporarily lowering ECMO flow rates can reduce the chance of air entering the circuit. Furthermore, maintaining adequate intravascular volume and applying appropriate positive pressure ventilation settings (such as positive end-expiratory pressure, or PEEP) can counteract negative pressures in the venous system that promote air entry.

The aim of this study was to simulate AE in the ECMO circuit. We investigated whether and under what circumstances the central venous catheter may be a potential entry point for air into the patient and the ECMO circuit and how different amounts of air affect ECMO performance at different pump speeds.

## 2. Methods

**Figure 1:**
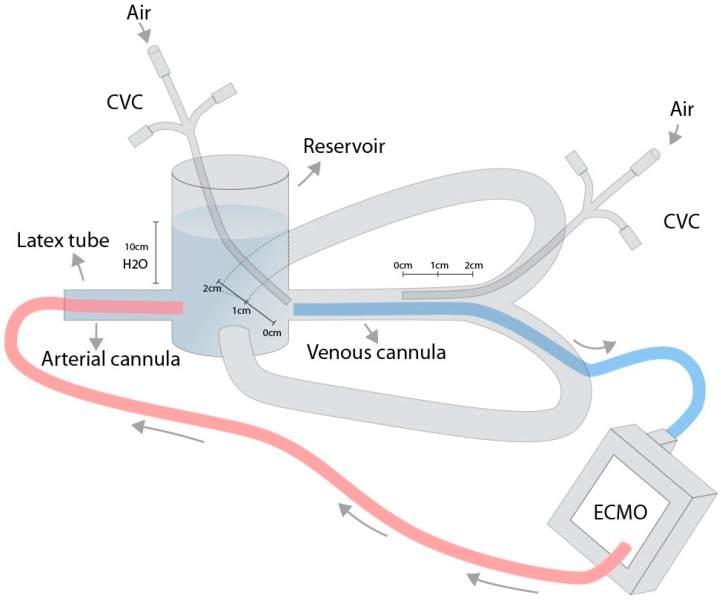
**Assembly of the model.** The model was constructed using standard 3/8 inch tubing made of latex, featuring an outer diameter of 12 mm, an inner diameter of 10 mm, and a wall thickness of 1 mm. This configuration was chosen to simulate vascular structures and facilitate cannulation. The tubing was securely attached to a plastic container, allowing for controlled manipulation of the fluid dynamics within the system. The assembly was designed to replicate the conditions encountered in an ECMO circuit.

An in vitro circulation model was constructed for the experiment (Figure 1). A 40% glycerol/water mixture at 36 °C was used, with fluid properties similar to those of blood [3]. The temperature of the mixture was maintained with an integrated heating unit (HU 35, Maquet, Getinge Group, Rastatt, Germany). A reservoir was filled to 10 cm to simulate a central venous pressure of 10 cm of water. Latex tubing with an inner diameter of 10 mm and a wall thickness of 1 mm was used to simulate vessels inserted at the bottom of the reservoir. Maquet Cardiohelp (Maquet, Getinge Group, Rastatt, Germany) and HLS Set advanced 7.0 (Maquet, Getinge Group, Rastatt, Germany) were used for ECMO. A multi-stage venous cannula (venous HLS cannula 23 Fr, 55 cm (Maquet, Getinge Group, Rastatt, Germany) and a single-stage arterial cannula (arterial HLS cannula 17 Fr, 15 cm (Maquet, Getinge Group, Rastatt, Germany) were inserted into the reservoir through latex tubing. The pressures at the tip of the central venous catheter were measured using a TruWave (3cc) VAMP Plus (215 cm) (Edwards Lifesciences, Irvin, CA, USA) pressure monitoring set and an IntelliVue MX750 (Philips, Amsterdam, Netherlands) patient monitor.

### 2.1. Air Entry Through Central Venous Catheter

Two CVCs (ARROWgard Blue PLUS^®^ Multi-Lumen CVC Set 7 Fr, 3 lumen, 20 cm (Arrow International, Reading, PA, USA)) were inserted into the system, one with a distal tip near the distal lumen and the other with a distal tip near the proximal side hole of the drainage cannula. The distance between the CVC tip and the hole in the drainage cannula was changed between experiments, with the distance between the CVC tip and the proximal or distal drainage hole being 0, 1, and 2 cm. The proximal CVC opening was at the same level as the drainage cannula and was either left open (unsecured), secured with a 3-way stopcock (Cair L.G.L, Lissieu, France), or secured with a clave (MicroClave, ICU Medical, Hannover, Germany). The experiment was performed at different pump speeds of 1500, 2500, 3500, and 4500 rpm. Air inflow through the CVC was monitored with an ultrasonic bubble detector integrated into the ECMO circuit at the drainage cannula. The system was completely deaired between each experiment. All experiments were repeated three times.

### 2.2. Influence of Air Quantity on ECMO Performance

The ECMO circuit was additionally modified with a side port on the venous cannula to allow controlled application of 1, 5, 25, and 50 mL of air with a syringe. Applications were performed at various pump speeds of 1500, 2500, 3500, and 4500 rpm. Airflow through the oxygenator was monitored with the ultrasound detector integrated into the ECMO system, which was attached to the return cannula. The ECMO system was observed for characteristic sounds (hissing sound, murmur), airflow through the oxygenator, and pump performance. The system was completely deaired between each trial. All experiments were repeated three times.

### 2.3. Algorithm for Preventing Air Entry

Preparation and Setup: Verify the functionality of ECMO components, inspecting cannulas, connectors, and tubing for any visible cracks or wear. Calibrate and check air bubble detectors for accurate functioning. Prime the ECMO circuit with saline or a priming solution to remove initial air, ensuring no residual bubbles remain.

Catheter Connection: Prepare the catheter connection by inspecting and securing all tubing connections to prevent leaks and positioning the catheter within the circuit to simulate clinical placement.

ECMO Circuit Initiation: Connect the ECMO circuit to the central venous catheter model, slowly purging any remaining air from both the catheter and ECMO connections using a saline flush. Securely fasten all connections and use visual inspections or imaging, if available, to confirm an air-free circuit. Activate the air bubble detectors for real-time alerts.

Monitoring During the Experiment: Conduct regular visual inspections along the tubing to identify any visible air bubbles, particularly at connection points. Employ continuous monitoring and bubble detectors to promptly detect any potential air entry. Carry out routine system checks periodically.

Air Detection Protocol: If air is detected, follow a troubleshooting and emergency response protocol. Temporarily halt simulated blood flow in the ECMO circuit, isolate the affected area, and aspirate the air if possible. Document each incident of air detection, including the location of air entry, estimated volume, and corrective actions taken.

### 2.4. Ethical Aspect

Since this was a simulation without the involvement of humans or animals, an ethical evaluation was not required.

### 2.5. Statistics

The results are presented in descriptive form. No statistical analysis was performed.

## 3. Results

**Figure 2:**
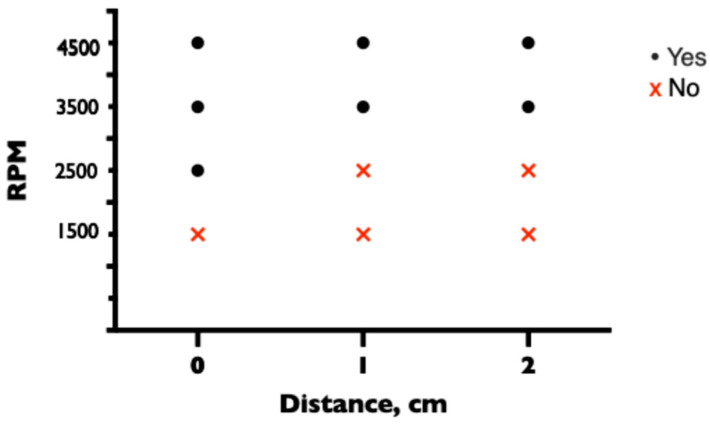
Entrance of air through unprotected lumen of CVC depending on pump speed and tip-to-tip distance.

When the CVC tip was positioned at the distal lumen of the ECMO drainage cannula, no air entered the ECMO circuit when the CVC lumen was protected with a three-way stopcock or clave at any of the tested pump speeds of 1500 (1.61 L/min), 2500 (3.15 L/min), 3500 (4.56 L/min), or 4500 (5.91 L/min) rpm and a prespecified tip-to-tip distance of 0, 1, and 2 cm. However, when the CVC lumen was left open, air entered the ECMO circuit at the pump speeds of 2500, 3500, and 4500 rpm when the tips were adjacent (tip-to-tip distance of 0 cm) but not at a pump speed of 1500 rpm. When the CVC lumen was left open and the tip-to-tip distance was increased to 1 or 2 cm, air entered the ECMO circuit only at the higher pump speeds of 3500 and 4500 rpm but not at the lower speeds of 1500 and 2500 rpm (Figure 2).

**Figure 3:**
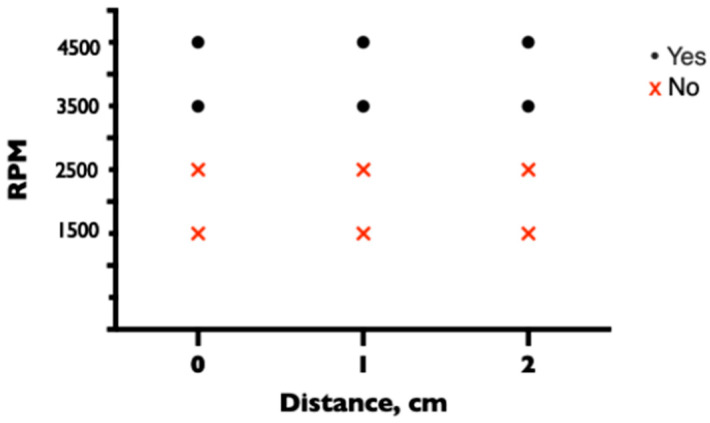
Entrance of air through unprotected lumen of CVC depending on pump speed and proximal side hole and tip distance.

When the tip of the CVC was positioned adjacent to the proximal side hole of the drainage cannula, the protection of the CVC with a three-way stopcock or clave was sufficient to prevent air entry at all pump speeds and positions. When the lumen of the CVC was left open, air entered the ECMO circuit at 3500 and 4500 rpm at all distances between the proximal side hole of the drainage cannula and the CVC of 0, 1, or 2 cm. At lower pump speeds of 1500 and 2500 rpm, no air entered through the unprotected lumen, regardless of the distance between the tips (Figure 3).

**Figure 4:**
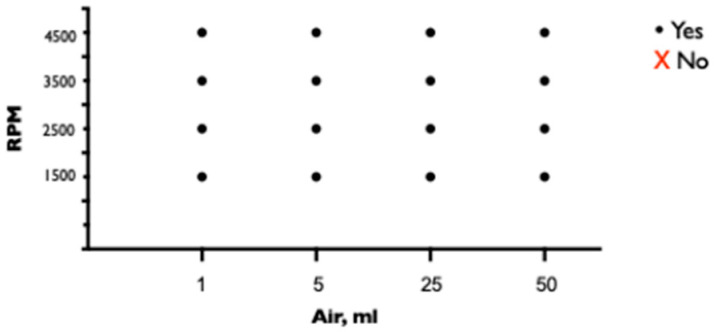
Presence of pump murmur after injection of air into ECMO circuit.

The characteristic sound of air in the pump is the first warning sign of air in the ECMO circuit and occurred at a minimum volume of 1 mL of air injected into the drainage cannula (and at all higher volumes) (Figure 4).

**Figure 5:**
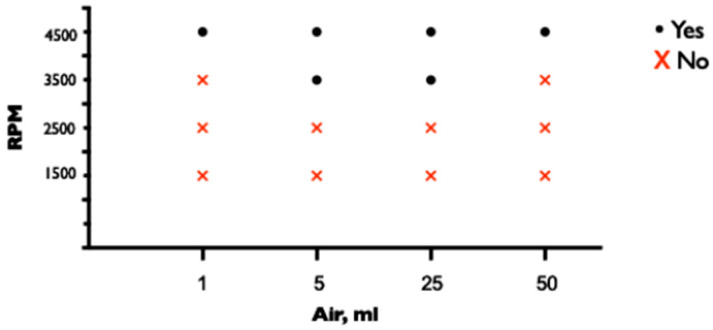
Passage of air trough membrane lung and activation of bubble detector alarm mounted on return cannula after injection of air into ECMO circuit.

Air passed through the oxygenator and triggered a bubble detection alarm at the return cannula when 1 mL of air was injected at 4500 rpm, 5 mL of air at 3500 and 4500 rpm, and 25 mL of air at 3500 and 4500 rpm (Figure 5).

**Figure 6:**
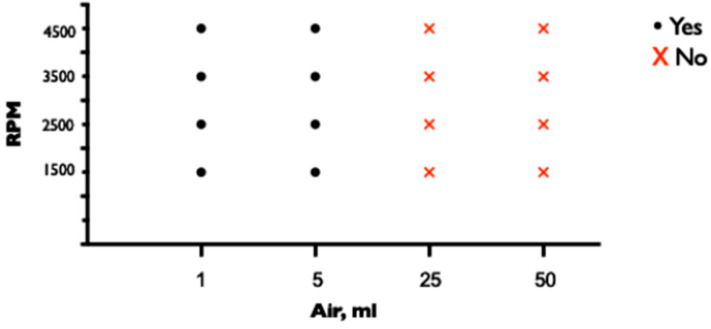
Complete pump stop after injection of air into ECMO circuit.

ECMO flows ceased completely when either 25 or 50 mL of air was injected into the drainage cannula at each of the pump speeds tested (Figure 6). Lower air volumes decreased flows for a short time, but the oxygenator slowly vented and regained flows.

## 4. Discussion

Our results confirm the possibility that air can enter the patient and the ECMO circuit through the unprotected lumen of the CVC, not only through the distal hole of the drainage cannula but also through the proximal side hole of the drainage cannula. As expected, the probability of air entry was higher when the distance between the hole in the drainage cannula and the tip of the CVC was smaller and the pump speed was higher. Air entry through the unprotected lumen of the CVC was more likely when the tip of the CVC was near the distal hole of the drainage cannula than when it was near the proximal hole of the drainage cannula.

Regardless of the distance between the CVC and the drainage cannula or the pump speed, a closed three-way stopcock and clave provide good protection against air emboli.

It is a common belief that mechanically ventilated patients are at decreased risk of air embolism from central venous catheters (CVCs) due to the protective effects of positive pressure ventilation and positive end-expiratory pressure (PEEP) compared to spontaneously breathing patients. However, our study demonstrates that, in the ECMO setting, the negative pressure generated by the drainage cannula can still lead to air entry into the circuit. This finding challenges the assumption that mechanically ventilated patients are inherently protected against air embolism, highlighting the unique risks associated with ECMO support.

The results of our research are supported by published examples from clinical practice. Kumar et al. describe a case in which the ECMO pump failed because of air entry through the open lumen of the CVC and even indicate a linear relationship between flow rates and the possibility of AE [7]. Air entry can be effectively prevented by appropriate measures such as the installation of a clave on the CVC. However, the installation of a clave is not a complete guarantee of safety, as air can also enter through the infusion system or during the administration of therapy if not handled carefully.

Another possible situation in which air can enter through the CVC is when the CVC is inserted. This could be prevented by careful planning and insertion of the CVC or other invasive procedures before starting ECMO therapy. The femoral position of the CVC might be the preferred position because there is less possibility of air entry through the proximal side hole of the drainage cannula.

If a CVC must be placed or an invasive procedure needs to be performed in a patient already on ECMO, the likelihood of air entry can be reduced by lowering flow rates through ECMO, ensuring adequate intravascular filling, and preventing spontaneous respiratory motion. If the CVC is in close proximity to the ECMO drainage cannula, it should be inserted at another site or withdrawn.

In our experiment, the characteristic pump noise was always present, regardless of the amount of air injected or the pump speed. We observed that the pump noise persisted longer at lower pump speeds but became louder as pump speed increased. Most of the time, the air was retained and removed by the membrane lung and did not enter the return cannula and trigger the bubble detector attached to the return cannula. However, at a pump speed of 4500, 1 mL of air entered the arterial portion of the ECMO system, indicating that a small amount of air may pass through the membrane into the arterial portion of the ECMO system at high pump speeds, also triggering the bubble detector alarm. As the amount of air increased, the likelihood of air passing through the membrane at lower pump speeds also increased. Therefore, a bubble detector attached to the return cannula is inappropriate for monitoring air in the circuit because smaller amounts of air are retained by the membrane lung. If a bubble detector is not attached to the drainage cannula, special attention should be paid to the pump noise, as it is the only accurate indication of air in the system in this particular case.

Larger volumes of air would temporarily disrupt pump performance and result in reduced flow. However, air volumes as low as 25 mL at lower pump speeds of 1500 rpm can completely stop the pump and ECMO support. At higher pump speeds, pump failure due to air entrapment is less likely, but air is more likely to enter the return cannula and pose an immediate risk to the patient. This finding is supported by data from previously published studies. For example, Lother et al. proposed that the risk of air entrapment is higher at higher ECMO pump blood flow rates [5].

Vieira et al. state that a larger volume of air prevents ECMO from working [8]. In reviewing their paper, we did not find the exact volume of air anywhere, but we can confirm this based on the results of our study. We found that as little as 25 mL of air displaces the blood from the oxygenator pump and causes the ECMO pump to stop completely. If the pump stopped completely, no more air would enter the return cannula. Increasing the pump speed risks restarting the pump and forcing air through the membrane lung, so the only way to avoid air embolism in the patient is to clamp the circuit.

From the results of our experiments, we can conclude that even a small amount of air entering the ECMO system is dangerous for patients treated with ECMO and carries the risk of systemic air embolism or interruption of ECMO support.

## 5. Study Limitations

The experiments were performed in vitro on a simulator. However, given the many parameters that influence ECMO performance, it is impossible to reproduce the interactions in vivo. Therefore, caution should be exercised when transferring the results to the patient.

A veno-venous configuration of the ECMO system was proposed for the experimental setup. Higher pressures on the return side in the case of a veno-arterial configuration could lead to different results. In addition, we experimented with only one size of CVC and only investigated the possibility of air embolism through the distal lumen and not through the side lumens of the CVC.

The different physical properties of the glycerol/water mixture used as blood substitute could be a limitation. Also, it was not possible to simulate the effect of respiration, which cyclically changes the pressure in the chest. With a more severe negative pressure in the chest, which may occur with vigorous inspiration, the potential for air to enter via the CVC would be much greater than with the central venous pressure of 10 cm H_2_O that we assumed.

## 6. Conclusions

Air embolism through the unprotected lumen of the CVC is possible not only when the tips of the distal lumen of the drainage cannula and the CVC are adjacent but also through the side hole of the drainage cannula. The likelihood of air embolism is greater at higher pump speeds and when there is less distance between the holes in the drainage cannula and the tip of the CVC. A closed three-way stopcock or clave on the CVC provides adequate protection against air embolism.

The characteristic sound of air in the pump is the first warning sign of air in the circuit. The passage of air through the ECMO membrane is more likely at higher pump speeds. As little as 25 mL of air entering the drainage cannula is enough to stop the pump and interrupt ECMO support.

## Data Availability

The datasets used and/or analyzed during the current study are available from the corresponding author on reasonable request.
